# The Neurovascular Properties of Dental Stem Cells and Their Importance in Dental Tissue Engineering

**DOI:** 10.1155/2016/9762871

**Published:** 2016-09-05

**Authors:** Jessica Ratajczak, Annelies Bronckaers, Yörg Dillen, Pascal Gervois, Tim Vangansewinkel, Ronald B. Driesen, Esther Wolfs, Ivo Lambrichts, Petra Hilkens

**Affiliations:** Laboratory of Morphology, Biomedical Research Institute (BIOMED), Hasselt University, Diepenbeek, Belgium

## Abstract

Within the field of tissue engineering, natural tissues are reconstructed by combining growth factors, stem cells, and different biomaterials to serve as a scaffold for novel tissue growth. As adequate vascularization and innervation are essential components for the viability of regenerated tissues, there is a high need for easily accessible stem cells that are capable of supporting these functions. Within the human tooth and its surrounding tissues, different stem cell populations can be distinguished, such as dental pulp stem cells, stem cells from human deciduous teeth, stem cells from the apical papilla, dental follicle stem cells, and periodontal ligament stem cells. Given their straightforward and relatively easy isolation from extracted third molars, dental stem cells (DSCs) have become an attractive source of mesenchymal-like stem cells. Over the past decade, there have been numerous studies supporting the angiogenic, neuroprotective, and neurotrophic effects of the DSC secretome. Together with their ability to differentiate into endothelial cells and neural cell types, this makes DSCs suitable candidates for dental tissue engineering and nerve injury repair.

## 1. Introduction

The main goal of tissue engineering is to reconstruct natural tissues by combining progenitor/stem cells with growth factors and different biomaterials to serve as a scaffold for novel tissue growth [1]. Selecting a suitable stem cell source is probably the most essential component of a successful tissue engineering approach. The field of tissue engineering is in need of high quality adult stem cells from an easily accessible source. Within the human body a wide variety of stem cell niches have been identified, not only in bone marrow, adipose tissue, and umbilical cord but also in teeth [2–6]. During tooth development, an outer layer of enamel and an inner layer of primary dentin are formed by reciprocal, spatiotemporal interactions between neural crest-derived mesenchyme and embryonic oral epithelium [7, 8]. Primary dentin is produced by odontoblasts, cells that are thought to arise from precursor cells residing in a strongly innervated and vascularized soft connective tissue within the tooth, that is, the dental pulp. In 2000, Gronthos et al. were the first to describe a heterogeneous, clonogenic, and highly proliferative cell population within the dental pulp, namely, dental pulp stem cells (DPSCs) [4]. A similar stem cell population could also be isolated from the dental pulp of human deciduous teeth [9]. In addition to DPSCs and stem cells from human exfoliated deciduous teeth (SHEDs), a number of other distinct stem cell populations have been reported to reside within the human tooth and its surrounding tissues. For example, stem cells from the apical papilla (SCAPs) can be found in the loosely attached soft connective tissue at the apex of developing permanent teeth, that is, the apical papilla [10]. Dental follicle stem cells (FSCs), on the other hand, are isolated from the dental follicle. This is a loose connective tissue which surrounds developing teeth and later on in development gives rise to the periodontal ligament and other tissues of the periodontium [11]. The periodontal ligament, a specialized connective tissue, not only attaches the tooth to the alveolar bone but also has a sensory function. Within this ligament, another stem cell population can be found, namely, periodontal ligament stem cells (PDLSCs) [12]. According to the minimal criteria defined by the International Society for Cellular Therapy, DPSCs, SHEDs, SCAPs, FSCs, and PDLSCs (collectively referred to as dental stem cells (DSCs)) are considered to be mesenchymal stem cells (MSCs). In addition to their plastic adherence and characteristic expression of surface markers such as CD73, CD90, and CD105, they also display a negative expression of CD14, CD34, and CD45, and they are capable of osteogenic, chondrogenic, and adipogenic differentiation [4, 13–15]. Next to the formation of dental tissue* in vitro* and* in vivo*, DSCs have also been reported to differentiate into myogenic, neurogenic, and endothelial lineages. Due to this multilineage differentiation potential as well as their immunomodulatory properties and minimally invasive isolation from extracted third molars, these stem cells have raised high hopes for potential clinical applications [16–21]. Nevertheless, one should always take into account potential origin-related differences. In general, SCAPs and FSCs are considered to be more immature, given their origin from developing dental tissues, and thus more potent in comparison to DPSCs. SCAPs have already been reported to have a higher proliferation rate, a more distinct doubling capacity, and enhanced migratory properties in comparison to DPSCs [10]. Furthermore, the glial origin of a subpopulation of DPSCs suggests that the tissue of origin is a determining factor for the regenerative potential of DSCs [22, 23]. In order to offer an elaborate overview of the angiogenic and neurogenic properties of different DSC populations as well as their current clinical applications in the dental and neurovascular field, a literature search was performed on PubMed. The following keywords were used: “dental stem cells”; “dental pulp stem cells”; “stem cells from the apical papilla”; “stem cells from human exfoliated deciduous teeth”; “dental follicle stem cells”; “periodontal ligament stem cells”. These keywords were subsequently combined with the search terms, “angiogenesis”; “endothelial differentiation”; “neurogenic differentiation”; “neuroregeneration”; “dental tissue engineering”; “dental pulp regeneration”; “periodontal regeneration”; “peripheral nerve injury”, without any set limitations regarding the type or year of publication.

## 2. Dental Stem Cells and Angiogenesis

Within the healthy human body, the most predominant and most studied form of blood vessel formation is angiogenesis. In general, angiogenesis can be defined as the sprouting of new capillaries from preexisting blood vessels in response to specific stimuli such as inflammation or hypoxia [42, 43]. This well-coordinated biological process is regulated by a broad range of proteins, which maintain a natural balance between stimulatory and inhibitory signaling pathways. As the latter are considered to be dominant, endothelial cells normally remain quiescent within the healthy human body [44]. However, in pathological conditions such as ischemic stroke, myocardial infarction, cancer, and diabetes, this balance is disturbed [45].

Since deprivation of oxygen and nutrients, due to a lack in vascular supply, can lead to tissue necrosis, angiogenesis also plays an important role in tissue engineering. However, the limited success of growth factor-based revascularization urged the need to promote angiogenesis with a more regenerative approach by means of stem cell-based therapies [46, 47]. MSCs are considered to establish therapeutic angiogenesis by either paracrine secretion of angiogenic growth factors or differentiation into endothelial cells [48–50].

### 2.1. Paracrine Mediation of Angiogenesis by Dental Stem Cells

With regard to the angiogenic properties of DSCs, studies have indicated the secretion of a broad range of regulatory proteins. DPSCs, for example, have been reported to express stimulatory growth factors such as platelet-derived growth factor (PDGF), basic fibroblast growth factor (bFGF), and vascular endothelial growth factor (VEGF), either in basal conditions or in response to noxious stimuli, for example, injury or hypoxia [17, 28, 29, 34, 35]. Other angiogenesis-promoting factors that have been detected in DPSCs are angiogenin (ANG), angiopoietin-1 (ANGPT1), colony-stimulating factor (CSF), dipeptidyl peptidase IV (DPPIV), endothelin-1 (EDN1), interleukin-8 (IL-8), insulin-like growth factor binding protein-3 (IGFBP3), monocyte chemoattractant protein-1 (MCP-1), and urokinase-type plasminogen activator (uPA) [24, 30]. Nevertheless, the secretome of DPSCs also comprises several inhibitory proteins, such as endostatin, pentraxin-3 (PTX-3), pigment epithelium-derived factor (PEGF), plasminogen activator inhibitor-1 (PAI-1), tissue inhibitor of matrix metalloproteinase-1/4 (TIMP-1/4), and thrombospondin-1 (THBS-1) [24, 30]. Comparable findings were also described for SCAPs and FSCs, albeit with variable expression levels between the different stem cell populations [16, 24–26, 31, 51]. With regard to the secretome of SHEDs and PDLSCs, literature indicates the expression of ANGPT2 [27], bFGF [16, 27, 32], endostatin [16], hepatocyte growth factor (HGF) [33], insulin-like growth factor-1 (IGF-1) [27], and VEGF [27, 32, 33, 36]. An overview of the DSC secretome and its associated functions can be found in Table 1 [44, 45, 52]. One should take into account the fact that not all growth factor functions have already been described for DSCs.

Since DSCs express a wide variety of angiogenesis regulating proteins, stimulatory as well as inhibitory, it is important to determine their potential impact on the behavior of endothelial cells and angiogenesis altogether. Each well-coordinated event within the angiogenic process can be mimicked by a series of* in vitro* assays. For instance, colorimetric assays are performed to evaluate the effect of DSC-derived growth factors on endothelial proliferation. A significant increase of both survival and proliferation of human umbilical vein endothelial cells (HUVECs) was observed after incubation with conditioned medium (CM) of a CD31^−^/CD146^−^ subpopulation of DPSCs [53]. Aranha et al. also reported a time-dependent increase in the proliferation of human dermal microvascular endothelial cells (HDMECs) when incubated with CM of hypoxia-preconditioned DPSCs [34]. Hilkens et al., on the other hand, reported no pronounced effect of CM of DPSCs, SCAPs, and FSCs on the proliferation of human microvascular endothelial cells (HMECs) [24]. To date, the potential effect of SHEDs and PDLSCs on endothelial proliferation has not been described. In order to evaluate whether endothelial cells migrate along a gradient of DSC-derived chemokines, a transwell migration assay is performed. DPSCs as well as SCAPs have been shown to significantly augment endothelial migration in comparison to FSCs [24, 30]. In terms of endothelial tubulogenesis, Yuan et al. indicated an increased formation of capillary-like structures during a direct coculture of SCAPs and HUVECs [54]. Similar outcomes were found for DPSCs, SHEDs, and PDLSCs [29, 32, 55–57]. During these direct cocultures, DSCs are thought to adopt a pericyte-like function as they are often found in close proximity to the endothelial cells [54, 56, 57]. Alternatively, endothelial tube formation can also be mediated by paracrine factors, as was shown by Dissanayaka et al. through an indirect coculture of DPSCs and HUVECs [58]. In line with these findings, Tran-Hung et al. and others reported a significant increase in endothelial tubulogenesis caused by CM of DPSCs [24, 29]. With regard to the impact of PDLSCs and SHEDs on the functional behavior of endothelial cells, more extensive research is required.

The angiogenic properties of DSCs have also been elaborately investigated* in vivo*. Yeasmin et al., for example, indicated significant vascularization after subcutaneous transplantation of PDLSCs and endothelial cells. Since there was no detection of human-derived blood vessels, PDLSCs were considered to secrete paracrine mediators or to act as pericytes [32]. Mouse DPSCs were found to induce angiogenesis in a VEGF-dependent manner in a mouse Matrigel plug assay [56]. DPSCs and SCAPs also caused a significant increase in angiogenesis in a chorioallantoic membrane assay [24, 30]. In terms of more clinically relevant disease models, Gandia et al. demonstrated a significant improvement of left ventricular function after injection of GFP-labeled DPSCs in a rat model of myocardial infarction. Apart from a reduction in infarct size and thickening of the anterior ventricular wall, an increase in capillary density was also detected. As there were no signs of differentiated DPSCs within the heart tissue, the aforementioned improvement was probably mediated by paracrine factors [59]. These findings were supported by Iohara et al., who reported a high capillary density after transplantation of a CD31^−^/CD146^−^ subpopulation of DPSCs in a mouse model of hindlimb ischemia. The close location of the stem cells near the newly formed blood vessels suggests a paracrine role for DPSCs [53]. The abovementioned subpopulation of DPSCs also promoted functional recovery in rats suffering from focal cerebral ischemia. Besides neurotrophic factors, the authors also demonstrated augmented levels of VEGF, which potentially played a role in the stimulation of vasculogenesis and neurogenesis in the ischemic rat brain [60, 61].

### 2.2. Endothelial Differentiation Potential of Dental Stem Cells

As stated before, MSCs not only contribute to therapeutic angiogenesis by secreting angiogenic growth factors but also are able to differentiate into endothelial cells under specific environmental cues. With regard to the endothelial differentiation potential of DPSCs, d'Aquino et al. were the first to report on the so-called codifferentiation of these cells into osteoblasts and endotheliocytes. Following the* in vitro* osteogenic differentiation of a sorted CD117^+^/CD34^+^/VEGFR2^+^ DPSC population, flow cytometric analysis demonstrated a subpopulation of VEGFR2^+^/Stro-1^+^/CD44^+^/CD54^+^ endothelial progenitor cells with a marked expression of von Willebrand factor (vWF) and angiotensin-converting enzyme (ACE) [62]. Similar results were found by Marchionni et al., indicating the expression of vWF and CD54 as well as the increased presence of VEGFR1 and VEGFR2, after incubating DPSCs with VEGF for 7 days. In addition, these cells were able to form capillary-like structures when seeded on Matrigel or cultured in a fibrin clot [63]. Extensive capillary network formation was also observed by Barachini et al., as well as the* in vitro* expression of CD31 and VEGFR2. Functionality of the differentiated cells was successfully established with the uptake of acetylated low density lipoprotein [64]. These findings confirm earlier observations made by Iohara et al., reporting the* in vitro* endothelial differentiation potential of a CD31^−^/CD146^−^ subpopulation of DPSCs [53]. However, the efficacy of endothelial differentiation is dependent on not only the addition of specific growth factors to the cell culture medium, but also the concentration of fetal bovine serum (FBS) and the cell seeding density appear to play an important role. Karbanová et al., for example, demonstrated the upregulation of CD31, CD34, CD106, and vWF after culturing DPSCs at a low density in serum-free differentiation medium. When cells were seeded at a higher density, no upregulation of vWF was observed. The addition of FBS maintained cell proliferation and the endothelial phenotype of DPSCs [65]. In addition to DPSCs, SHEDs have also been shown to differentiate into endothelial cells. In 2008, Cordeiro et al. detected beta-galactosidase-positive capillaries in transplanted tooth slices containing LacZ-transduced SHEDs [66]. The same researchers later on confirmed these results* in vitro* and* in vivo*, indicating capillary sprouting and the VEGF-induced expression of VEGFR2, CD31, and VE-cadherin and a continuous expression of VEGFR1 by SHEDs [67, 68]. In particular VEGFR1 appears to play an important role in the endothelial differentiation potential of SHEDs and DPSCs. This was demonstrated by a reduction of human CD31-positive capillaries, following transplantation of VEGFR1-silenced SHEDs in a tooth slice model* in vivo*. The importance of the VEGFR1/MEK1/ERK signaling cascade was illustrated* in vitro* by the complete suppression of endothelial differentiation following the inhibition of this signaling pathway [67]. Recent work of Zhang et al. also revealed the Wnt/*β*-catenin pathway to be an important regulator of the endothelial fate of DPSCs and SHEDs [69]. With regard to the endothelial differentiation potential of other DSC populations, limited data are available. Bakopoulou et al. recently demonstrated the acquisition of a preendothelial phenotype by SCAPs, after exposure to an angiogenic induction medium for 28 days in normoxic conditions. These cells not only were able to form capillary-like structures but also displayed a time-dependent upregulation of different marker proteins, such as CD31, vWF, VEGFR2, angiopoietin-1/2, and Tie-1. Moreover, when depriving SCAPs of oxygen and nutrients, their endothelial differentiation potential appeared to be more pronounced [26]. After induction of differentiation for different periods of time, both a significant upregulation of endothelial marker proteins and the formation of tubules were observed in a CD105^+^-enriched subpopulation of PDLSCs. Molecular analysis illustrated the critical role of neuropilin-2 (NRP-2) in the angiogenic fate of these stem cells [70]. To date, evidence for the* in vivo* endothelial transdifferentiation of DSCs remains scarce. As previously mentioned, Zhang and colleagues observed the presence of human CD31^+^ blood vessels after transplanting human DPSCs and SHEDs in a rodent tooth slice model [67, 69]. However, DSCs are mainly considered to assume a pericyte-like phenotype, as they are often located adjacent to endothelial cells* in vitro* as well as* in vivo* [32, 54–57, 71, 72].

## 3. Dental Stem Cells and Neuroregeneration

### 3.1. Paracrine Mediation of Neuroprotection and Neurite Outgrowth by Dental Stem Cells

DSCs also produce a wide variety of neurotrophic factors and therefore they can be used in tissue engineering as a growth factor delivery system. These neurotrophic factors play a pivotal role in protecting neurons from apoptosis and inducing endogenous neural repair and neurite formation. Brain-derived neurotrophic factor (BDNF), neurotrophin-3 (NT-3), glial cell-derived neurotrophic factor (GDNF), nerve growth factor (NGF), and various others are abundantly present in the secretome of DPSCs and SCAPs (Table 1) [25, 38, 40, 41, 73–77]. The neurotrophic factors secreted by SHEDs, PDLSCs, or FSCs remain to be characterized. Mead et al. demonstrated that DPSCs expressed more NGF, BDNF, and VEGF than bone marrow-derived mesenchymal stem cells (BM-MSCs) and adipose tissue-derived mesenchymal stem cells (AMSCs) [40]. Another research group revealed that SCAPs secreted significantly larger amounts of chemokines and neurotrophins in comparison to BM-MSCs, whereas BM-MSCs secreted more extracellular matrix (ECM) proteins and proangiogenic factors [25]. Interestingly, the capacity of DPSCs to increase neurite outgrowth of neurons of dorsal ganglia was even more pronounced after these cells were differentiated into Schwann cells [38]. In addition, another study showed that after differentiation into neurons, DPSCs expressed more VEGF and NGF but less BDNF [78].

In comparison to other DSC populations, there is abundant evidence on the beneficial effect of DPSCs on neuroprotection and neuritogenesis* in vitro*. DPSCs were found to rescue sensory and dopaminergic neurons from apoptosis [79] and induce the survival and sprouting of neurons of trigeminal [75], retinal [40, 80], and sympathetic ganglia [38, 81]. SHED CM also enhances the viability and neuritogenesis of neurons of dorsal root ganglia [82]. A recent paper showed that exosomes derived from SHEDs grown on laminin-coated three-dimensional alginate microcarriers are able to suppress 6-hydroxy-dopamine-induced apoptosis in dopaminergic neurons [83]. DPSCs significantly enhanced neuritogenesis of axotomized rat retinal ganglia compared to BM-MSCs and AMSCs and possessed superior neuroprotective properties. The addition of specific Fc-receptor inhibitors revealed that VGF nerve growth factor inducible (VGF) was the responsible factor released by DPSCs [40]. Finally, DPSCs were shown to be superior to BM-MSCs in rescuing astrocytes from cell death induced by oxygen-glucose deprivation [84]. DPSCs are also able to guide the differentiation process of neural precursor cells: rat neural stem cells cultured on P(EA-co-HEA)90/10 biomaterials covered with DPSCs differentiated into young Tuj1-immunoreactive neurons [73].

Numerous studies have demonstrated the beneficial effects of DPSCs in injuries and pathologies of nervous system. In the pioneer study of Arthur et al., DPSCs were able to attract trigeminal neurons after transplantation into chicken embryos [76, 81]. As mentioned above, neurogenic predifferentiated DPSCs were also able to integrate in the host brain of a rat [85]. Intravitreal injection of DPSCs promotes neuronal survival and axon regeneration of retinal ganglia cells after optic nerve injury in rats [39]. In a rat model of spinal cord injury, transplantation of both SHEDs and DPSCs improved recovery of hindlimb locomotor functions. In the same experiment, BM-MSCs or skin-derived fibroblasts caused substantially less recovery of locomotor function. The proposed mechanisms were inhibition of apoptosis of neurons, astrocytes, and oligodendrocytes and regeneration of transected axons [86]. In rodent models of ischemic stroke, transplantation of DPSCs and SHEDs also led to an improvement in neurobehavioral function [61, 87–90]. This could be due to a decrease of inflammation, an increase of angiogenesis, or a reduction of apoptosis. Nagpal et al. announced the first clinical trial to apply autologous DPSCs as a therapy for patients with chronic disability after stroke, the so-called TOOTH trial (The Open study Of dental pulp stem cell Therapy in Humans, TOOTH) [91]. Both SHEDs and DPSCs differentiated to dopaminergic neurons have been shown to improve functional behavior in a rat model of Parkinson's disease. The therapeutic success was attributed to the induction of neurite outgrowth and neuronal survival caused by various neurotrophic factors [92]. Very recently it was described that a single injection of SHED CM in mice suffering from experimental autoimmune encephalomyelitis not only significantly improved disease scores and reduced demyelination and axonal injury but also reduced inflammatory cell infiltration and proinflammatory cytokine levels* in vivo* [93].

Studies describing the neuroprotective effects of FSCs, SCAPs, or PDLSCs are scarce. In a coculture system with rat trigeminal ganglia, SCAPs were able to stimulate and guide neurite outgrowth* in vitro*. This effect was completely inhibited by neutralizing antibodies directed against BDNF but no effect was observed when NGF and GDNF were blocked. In addition, axonal growth was shown to be triggered after subcutaneous injection of a Matrigel containing SCAPs into immunodeficient mice [37]. Furthermore, SCAPs were also applied in a rat model of spinal cord injury. However, the functional outcome was better in animals which received implantation of a whole apical papilla compared to animals which received* in vitro* expanded SCAPs [94]. FSCs seeded on aligned electrospun poly(*ε*-caprolactone)/poly-DL-lactide-co-glycolide fibers were also applied as a cellular therapy in a spinal cord injury model, but no significant functional improvement was observed after transplantation [95].

### 3.2. Neuronal and Neural Differentiation Potential of Dental Stem Cells

As DSCs are embryonically derived from the neural crest and glial tissues, it is not surprising that these cells display neurogenic properties. Multiple protocols are available in literature to induce differentiation of DSCs into neurons* in vitro*. These procedures involve either the incubation of DSC monolayers with a cocktail of various growth factors and pharmacological compounds or the generation of floating neurospheres of DSCs, which recapitulates the embryonic stages of neuroblast formation (for a detailed review, see [74, 96]). In general, the mostly applied proteins to induce neuronal differentiation of DSC monolayers include epidermal growth factor (EGF) and bFGF in combination with culture supplements such as B27, forskolin, and insulin-transferrin-sodium selenite (ITS). All DSC populations have been shown to differentiate into neuron-like cells. Although successful differentiation was verified by means of an increased expression of neuronal markers such as NeuN, neural cell adhesion molecule, neurofilament, synaptophysin, A2B5, and microtubule-associated protein-2, ultrastructural and/or electrophysiological analysis of the differentiated cells was lacking in most of these studies. Furthermore, these differentiation protocols mostly result in a low yield of neurons, which are primitive, immature, and not able to produce a train of action potentials [78]. However, studies of Király et al. demonstrated that predifferentiated DPSCs integrated into the host brain when transplanted into the cerebrospinal fluid of 3-day-old rats with a cortical injury. These cells displayed neuronal properties, not only by expressing neurofilament and NeuN but also by exhibiting voltage-dependent sodium and potassium channels [85]. DPSCs that were injected into the brain of rodents after stroke predominantly differentiated into astrocytes instead of neurons [87]. As DSCs represent a very heterogeneous stem cell population, sorting the cells prior to neuronal induction might increase the success rate of differentiation.

Recent evidence indicates that DSCs also differentiate into oligodendrocytes and Schwann cells, which myelinate neurons from the central and peripheral nervous system, respectively. Transfection of the helix-loop-helix transcription factor Olig2 in DPSCs resulted in an increased expression of oligodendrocyte markers such as nestin, NG2, and myelin basic protein [97]. In addition, Martens et al. reported the differentiation of DPSCs into Schwann cells. The differentiated cells displayed an increased expression of laminin, low-affinity nerve growth factor receptor p75, glial fibrillary acidic protein, and CD104. Moreover, these cells were able to myelinate neurons* in vitro*, which was confirmed by ultrastructural analysis [38]. The ability of DPSCs to differentiate into Schwann cells might be explained by the fact that a significant population of the DPSCs are embryonically derived from peripheral nerve-associated glia [22] and might thus represent a dedifferentiation. Whether other DSCs are capable of differentiating into myelinating cells remains to be investigated. Because of their capacity to differentiate into Schwann cells, DPSCs might represent a promising strategy to treat peripheral nerve injury. Nerve autografts are the current gold standard treatment in the clinic. As this involves sacrifice of other nerves and the clinical results are usually unsatisfactory, other options are currently under investigation [98]. Despite their key role in endogenous nerve repair, transplantation of Schwann cells themselves is very restricted as their isolation also requires destruction of another nerve and their expansion rates are dramatically low [98–100]. In a rat model of facial nerve injury, DPSCs were shown to promote remyelination, blood vessel formation, and nerve regeneration when applied in combination with silicon, collagen I, or poly(lactic-co-glycolic acid) (PLGA) tubes [101–103]. SHEDs were also applied to treat rat sciatic nerve injury. Bridging the nerve gap with silicon conduits containing CM of SHEDs resulted in a higher number of myelinated axons and better functional recovery compared to silicon conduits containing control medium [82]. Finally, one study reported the use of PDLSCs in peripheral nerve injury. Injection of PDLSCs into the crush-injured left mental nerve of rats significantly improved sensory function and increased the number or retrograde labeled sensory neurons and myelinated axons [104].

Despite the fact that under controlled circumstances DSCs are able to differentiate into cells resembling to neurons, Schwann cells, and oligodendrocytes, the current paradigm is that their beneficial effects in preclinical models of neurodegenerative diseases and traumas are caused by the cytokines and growth factors in their secretome.

## 4. Preconditioning of Dental Stem Cells to Enhance Their Angiogenic and Neurogenic Properties

One of the major hurdles in the field of tissue engineering is the survival of transplanted cells* in vivo*. To overcome this obstacle, several strategies have been developed to modulate the stem cells prior to transplantation in order to improve cellular survival and engraftment [105]. Genetic modification offers a potential strategy to increase stem cell survival, for example, by overexpressing antiapoptotic genes such as Bcl-2 [106] or Akt [107, 108]. Another possibility is to modify the expression of a key protein of a certain illness such as dopamine for Parkinson's patients or insulin for diabetics [109]. However, genetic modification is a new and developing field and many questions remain to be resolved before clinical applications using genetically modified stem cells can be deemed possible [109].

Preparing stem cells for transplantation by exposing them to a hypoxic environment may be a useful technique to improve the stem cell secretome, since hypoxia is a potent stimulus for the secretion of a variety of trophic factors (Table 2) [105, 124]. Hypoxic preconditioning has already been shown to ameliorate cell survival, paracrine activity, and angiogenic potency in a model of murine hindlimb ischemia [125–127]. Oxygen levels in the dental pulp are lower compared to other tissues, since oxygen can only be supplied via blood vessels running through the rather narrow apical foramen of the tooth [128]. Culturing DPSCs under hypoxic conditions increases their proliferation rate [110, 111], VEGF expression [34], and migration [112]. Moreover, hypoxia also upregulates VEGF production in SCAPs [26, 31] and cells from the periodontal ligament [118]. These reports all support the beneficial effects of hypoxic preconditioning. However, mimicking hypoxia by simply adding a pharmacological agent would greatly increase the feasibility of this approach (Table 2). Prolyl hydroxylase (PHD) inhibitors represent such a group of hypoxia mimicking agents. PHD inhibitors include not only iron chelators such as hinokitiol, deferoxamine, or L-mimosine but also cobalt chloride and dimethyloxalylglycine [119, 129]. These PHD inhibitors promote VEGF secretion and HIF-1*α* expression in dental pulp-derived cells [113, 114], SCAPs [123], and PDL cells [119] and even in a tooth slice organ culture model [130]. Furthermore, preconditioned DPSCs [115] and SCAPs [123] also enhance capillary network formation by HUVECs. In addition, the application of hinokitiol-stimulated DPSCs in a mouse Matrigel plug assay resulted in an increased hemoglobin content and PECAM-1 expression [114]. Taken together, these reports suggest a promising future for the use of hypoxic mimicry in preparing stem cells for* in vivo* transplantation. HIF-1*α* and its downstream targets stimulate not only angiogenesis but also neurogenesis. For this reason, hypoxic preconditioning offers new prospects with regard to neuroregeneration. Despite the promising results using BM-MSCs [131–133] and embryonic stem cells [134], no reports were found using preconditioned DSCs for the treatment of neurological disorders.

To conclude, there are several more cytokines, growth factors, or chemical agents that can be used to boost the angiogenic potential of DSCs (Table 2). For example, bacterial lipopolysaccharide (LPS) has been shown to enhance VEGF secretion of DPSC [116, 117] and the migration of FSCs [122]. Pretreatment of PDLSCs with IL-1*α* [36] and TNF-*α* [120] leads to a more pronounced VEGF secretion, whereas adiponectin exposure increases PDLC proliferation rate and wound healing capabilities [121].

## 5. Dental Stem Cells and Pulp Regeneration

Although dental pulp can be characterized as a specialized tissue with a number of important physiological functions, it is also very vulnerable to caries, infections, and trauma. As any of these insults can easily interfere with normal pulp homeostasis and subsequently affect normal root development, the endodontic treatment of necrotic, immature permanent teeth in particular poses many challenges [135–137]. Over the past decade, substantial advances have been made regarding the potential application of DSCs in the regeneration of viable dental tissues. Not only has successful dental pulp regeneration been reported for DPSCs, but also SCAPs and FSCs have proven to be effective in different* in vivo* models of pulp regeneration.

Even before the definition and characterization of DPSCs, Mooney et al. already demonstrated the establishment of pulp-like tissue* in vitro* when culturing human pulp fibroblasts onto polyglycolic acid (PGA) matrices for 60 days [138]. In line with these findings, Buurma et al. reported fibroblast survival and ECM production within the PGA constructs after subcutaneous transplantation in immunocompromised mice [139]. Around the same time, Gronthos et al. and others described the presence of a stem cell population within the dental pulp, namely, DPSCs, which was able to form a vascularized dentin/pulp-like complex* in vivo* when cotransplanted with hydroxyapatite/tricalcium phosphate particles (HA/TCP) in immunocompromised mice [4, 14, 140]. These findings led to the development of other proof-of-principle models such as the tooth slice/scaffold model, which comprises the application of an emptied human tooth slice containing a supportive scaffold [141]. A number of studies have pointed out the regeneration of vascularized pulp-like tissue after subcutaneous implantation of tooth slices containing either DPSCs or SHEDs supported by a biodegradable scaffold [66, 67, 69, 141, 142]. Another proof-of-principle model, which illustrates the limited vascular supply within the tooth, is the ectopic root transplantation model. In 2010, Huang et al. described the* de novo* formation of a vascularized dentin/pulp complex in a subcutaneously transplanted emptied human root canal enclosing a PLGA scaffold seeded with DPSCs [143]. Similar observations were made by Rosa et al., showing the formation of vascularized dentin/pulp-like tissue after subcutaneous implantation of SHEDs and a self-assembling peptide hydrogel in emptied human root canals [144]. A specific, granulocyte colony-stimulating factor- (G-CSF-) mobilized subpopulation of DPSCs was also found to regenerate vascularized pulp tissue in an ectopic root transplantation model after a short 21-day transplantation period [145]. More recently, Dissanayaka et al. demonstrated the successful regeneration of vascularized dental pulp tissue after transplantation of DPSCs or DPSCs and HUVECs encapsulated in a commercially available hydrogel. Root fragments containing cocultures displayed a more pronounced vascularization, ECM deposition, and tissue mineralization in comparison to DPSCs alone, indicating the importance of coordinated cell-cell interactions [58]. Preconditioning DPSCs by means of hypoxia, prior to transplantation, evoked a higher number of blood vessels in the regenerated tissue compared to the control conditions [146]. Although the ectopic root transplantation model has been widely applied, both the shape of the root canals and the implemented size of the apical foramen have been prone to variability [58, 143–146], which leads to the question whether smaller apical openings would interfere with normal tissue regeneration in the “coronal” part of the emptied root canal [143]. Another important aspect which definitely needs to be taken into account during differentiation and tissue engineering is the specific microenvironment at the time of regeneration. This would require the* in situ* transplantation of DPSCs in (partially) pulpectomized teeth in (larger) animal models. In 2004, Iohara et al. reported the formation of reparative dentin after treatment of an amputated canine pulp with an autologous DPSC pellet incubated with bone morphogenetic protein-2 [147, 148]. Over the past decade this research group and others have successfully performed* in situ* transplantations (of different subpopulations) of DPSCs to completely regenerate vascularized pulp tissue [149–156]. In 2013, the first solid steps towards the clinical application of DPSCs were taken. After careful karyotyping and excluding potential tumor formation by the stem cells, Iohara et al. reported the regeneration of a vascularized and innervated dentin/pulp complex following transplantation of a clinical-grade subpopulation of DPSCs in pulpectomized canine teeth with an apical opening of 0.6 mm [152, 157].

With regard to the regenerative potential of SCAPs, Sonoyama et al. demonstrated their ability to form a dentin/pulp complex when transplanted with HA/TCP particles in immunocompromised mice. The human origin of the dentin-producing cells suggested the differentiation of SCAPs into odontoblast-like cells [158]. Similar results were also found by Huang et al., indicating the* de novo* regeneration of vascularized pulp-like tissue after the ectopic transplantation of an emptied human root canal containing a PLGA scaffold seeded with SCAPs [143]. Moreover, analysis showed a more continuous and thicker layer of dentin matrix in comparison to similar constructs containing DPSCs [143]. Subcutaneous implantation of SCAP-based cell sheet-derived pellets in immunodeficient mice also led to the development of a vascularized dentin/pulp complex with a continuous layer of dentin matrix [159].

As previously mentioned, FSCs originate from the dental follicle, a loose connective tissue surrounding developing teeth [11]. As the dental follicle during tooth development gives rise to the periodontium, research has mainly focused on the ability of FSCs to regenerate cementum and periodontal ligament [160–167]. Regarding their ability to form dental pulp tissue, Guo et al. indicated the establishment of an odontoblast-like cell layer as well as the formation of (pre)dentin in the omental pouch of adult rats after transplantation of rat FSCs and treated dentin matrix (TDM) [168]. In an attempt to engineer a complete tooth root, transplantation of similar constructs containing rat or human FSCs led to the regeneration of a dentin/pulp complex as well as cementum and periodontal ligament (PDL) [169, 170]. In line with these findings, Jiao et al. described the formation of vascularized dental pulp-like tissue after subcutaneous transplantation of human FSCs encapsulated within cryopreserved TDM [171]. When comparing the regenerative potential of FSCs and SCAPs, no significant differences were found despite their differential expression of protein markers* in vitro*. Both stem cell populations were able to regenerate a vascularized dentin/pulp-like complex following 8 weeks of transplantation in nude mice [51]. To date, no reports are available regarding the use of PDLSCs in dental pulp regeneration. Given their developmental origin, these stem cells have been mainly investigated for their potential application in periodontal regeneration.

## 6. Clinical Application of DSCs and Its Challenges

Despite the promising outcomes of DSC transplantation in a preclinical setting, the progression of DSCs from bench to bedside still holds some major challenges. Standardized cell isolation procedures, for example, are indispensable to safeguard the clinical safety, reproducibility, and efficacy of DSC therapy [172]. However, the extraction of third molars as well as the isolation of DSCs is currently being performed using diverse isolation procedures on donors of different ages with molars at different stages of development, which not only impairs in-depth comparison of experimental outcomes but also hinders the development of a standardized treatment protocol [15, 23, 173, 174]. Next to consistent isolation procedures, the clinical implementation of DSCs also entails the upscale production of these stem cells in xeno-free culture conditions, in order to provide an adequate amount of cells without any contamination of potential infectious agents [175–177]. Nevertheless, due to the inherent batch-to-batch variety as well as the current lack of reliable study protocols regarding the use of human blood-derived products as a potential alternative, more research is required before any educated decision can be taken by both scientists and regulatory agencies [178–180]. In addition to the challenges associated with the processing and culturing of DSCs, one also needs to take into account the intrinsic behavior of the stem cells, as it can be influenced by a broad range of different donor-related factors, such as (oral) health, age, and orthodontic tooth movement [23].

Although numerous studies have elaborately described the immunomodulatory effects of DSCs* in vitro*, little is known concerning the effects of allogeneic DSC transplantation* in vivo* [19, 181–189]. Tomic et al., for example, reported the formation of granulomatous tissue after xenogeneic transplantation of human DPSCs and FSCs in immunocompetent mice [190]. When transplanting rat DPSCs in mice suffering from colitis, on the other hand, a clear reduction of inflammation was observed [191]. There were also no signs of immune rejection after injection of human SHEDs in a canine model of muscular dystrophy [192]. In line with these findings, conditioned medium of SHEDs was found to alleviate autoimmune encephalomyelitis as well as to improve the cognitive function in a mouse model of Alzheimer's Disease through the induction of anti-inflammatory M2-phenotype microglia [93, 193]. Nevertheless, the outcome of allogeneic DSC transplantation for dental tissue engineering purposes in particular remains largely unknown, as most ectopic transplantation models are performed in immunocompromised mice and most* in situ* models apply autologous DSCs [58, 143–158, 162, 170, 194–197]. More research is thus required with respect to the immunomodulatory behavior of allogeneic DSCs* in vivo* and potential graft-versus-host responses.

When making the switch from bench to bedside it is also important to include sufficient patient-centered outcomes. All too often, dental clinical trials focus on technical, clinician-centered outcomes instead of patient-centered outcomes. Developing a standardized set of core outcomes could help overcome this fixation with clinician-based outcomes and lead to more consistent study designs [198].

Despite these challenges, a few clinical studies using DSC-based therapies are currently recruiting participants (Table 3). In India, a clinical study is currently ongoing in which patients suffering from chronic periodontitis receive a local injection of allogeneic human DPSCs in order to improve periodontal tissue regeneration (ClinicalTrials.gov NCT02523651). Allogeneic DPSCs are also being applied in a clinical trial in China, investigating the effect of DPSCs on osseointegration of dental implants (NCT02731586). Also in China, a second clinical trial focuses on the revitalization of young immature permanent teeth with necrotic pulps using autologous SHEDs (NCT01814436). Finally, Nagpal et al. announced a study protocol for evaluating safety and feasibility of autologous DPSC-based stem cell therapy in patients with chronic disability after stroke; however, this study is not yet recruiting participants [91].

## 7. Conclusion and Future Perspectives

**Figure 1:**
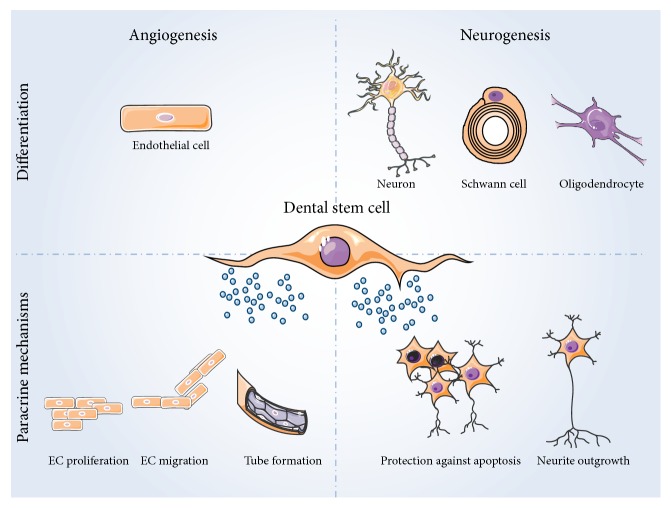
General overview of the angiogenic and neurogenic potential of DSCs. DSCs can differentiate into endothelial cells, neurons, Schwann cells, and oligodendrocytes under specific environmental clues. More relevant for their clinical applications is the fact that DSCs have a secretome rich in proteins which have a beneficial effect on surrounding cells. DSCs secrete a wide variety of angiogenic factors, inducing endothelial cell proliferation, migration, tube formation, and thus blood vessel development. DSCs also express neurotrophic factors which protect neurons from apoptosis and induce neurite outgrowth. This figure was made with images from the Servier Medical Art by Servier.

Taken together, DSCs are considered suitable candidates for cell-based treatment strategies and tissue engineering applications. There is abundant evidence supporting the angiogenic, neuroprotective, and neurotrophic actions of the DSC secretome (Figure 1). These inherent properties can even be augmented by pretreating DSCs prior to their transplantation. In particular hypoxia and hypoxia mimicking agents show great potential to improve stem cell survival and boost the DSC secretome. Up until now, most of the pretreatment studies have been focusing on improving the angiogenic effects of DSCs; therefore more research into the possible enhancement of their neurotropic/neuroprotective properties is warranted. In addition to their paracrine effects, DSCs have also been described to have the ability to differentiate into endothelial cells as well as neural cell types (Figure 1). Unfortunately, a wide variety of differentiation protocols have been used, resulting in highly variable outcomes and making it difficult to compare study outcomes but even more so to compare different DSC populations. Furthermore, often different parameters are used to assess successful differentiation.

Based on their origin, DSCs are expected to be ideal candidates for the regeneration of dental tissues such as the dental pulp and the periodontal ligament. Successful dental pulp regeneration has already been reported for DPSCs, SCAPs, and FSCs, whereas PDLSCs hold great potential for the regeneration of periodontal tissues. Furthermore, DPSCs, SHEDs, and PDLSCs have already been reported to improve regeneration after peripheral nerve injury by promoting remyelination, blood vessel formation, and nerve regeneration. These encouraging results contributed to the approval of two clinical studies that are currently recruiting participants and are thereby taking the first steps to introducing DSC-based therapies into the clinic.

## Figures and Tables

**Table 1:** The secretome of dental stem cells and its associated functions.

Factor	Function	Population	Reference
*Angiogenesis-stimulating factors*			

Angiogenin (ANG)	Endothelial proliferation and migration. Activation of smooth muscle cells. Indirect degradation of basement membrane.	DPSCs, SCAPs, and FSCs	[24]

Angiopoietin-1 (ANGPT1)	Endothelial survival, migration, and matrix adhesion. Endothelial sprouting and vessel stabilization.	DPSCs, SCAPs, and FSCs	[24–26]

Angiopoietin-2 (ANGPT2)	Endothelial proliferation, migration, and sprouting in the presence of VEGF.	PDLSCs	[27]

Basic fibroblast growth factor (bFGF)	Endothelial proliferation, migration, and tube formation. Upregulation of uPA, VEGF receptor, and integrins.	DPSCs, SCAPs, SHEDs, and PDLSCs	[16, 26–32]

Colony stimulating factor (CSF)	Endothelial proliferation, migration, and differentiation. Induction of proteolytic enzyme release.	DPSCs	[17]

CXC chemokines, for example, interleukin-8 (IL-8)	Endothelial survival, proliferation, migration, and tube formation. Induction of MMP production.	DPSCs	[30]

Dipeptidyl peptidase IV (DPPIV)	Vascular remodeling. Endothelial proliferation, migration, and tube formation.	DPSCs, SCAPs, and FSCs	[24]

Endothelin-1 (EDN1)	Endothelial proliferation and migration. Stimulation of VEGF-mediated angiogenesis. Stimulation of endothelial MMP2 production. Proliferation of vascular smooth muscle cells.	DPSCs, SCAPs, and FSCs	[24]

Hepatocyte growth factor (HGF)	Endothelial proliferation, migration, and tube formation. Proliferation of vascular smooth muscle cells. Stimulation of VEGF and PlGF production.	SCAPs, SHEDs	[25, 26, 33]

Insulin-like growth factor-1 (IGF-1)	Endothelial proliferation, migration, and tube formation. Stimulation of VEGF and plasminogen activator production. Downregulation of endothelial apoptosis.	PDLSCs	[27]

Insulin-like growth factor binding protein-3 (IGFBP3)	Endothelial migration and tube formation. Stimulation of IGF-1-mediated angiogenesis. Stimulation of VEGF and MMP2 production.	DPSCs, SCAPs, and FSCs	[24, 26, 30]

Matrix metalloproteinases (MMPs)	Extracellular matrix degradation and release of sequestered growth factors.	DPSCs, SCAPs	[17, 25]

Monocyte chemotactic protein (MCP-1)	Endothelial chemotaxis, tube formation, and differentiation. Stimulation of HIF-1*α* and VEGF production.	DPSCs, SCAPs	[26, 30]

Platelet-derived growth factor (PDGF)	Endothelial proliferation, migration, and differentiation. Stimulation of VEGF expression. Proliferation of vascular smooth muscle cells and pericytes. Vessel stabilization.	DPSCs	[28]

Urokinase-type plasminogen activator (uPA)	Participation in ECM degradation and release of sequestered growth factors. Endothelial migration and invasion. Activation of VEGF and pro-HGF.	DPSCs, SCAPs, and FSCs	[24, 26, 30]

Vascular endothelial growth factor (VEGF)	Endothelial proliferation, migration, and tube formation. Stimulation of NO synthase and plasminogen activator expression. Downregulation of endothelial apoptosis.	DPSCs, SCAPs, FSCs, SHEDs, and PDLSCs	[17, 24–36]

*Angiogenesis-inhibiting factors*			

ANGPT2	Natural antagonist of ANGPT1. Upregulation of endothelial apoptosis.	PDLSCs	[27]

DPPIV	Inhibition of endothelial progenitor homing. Inhibition of CXCR3-induced chemotaxis.	DPSCs, SCAPs, and FSCs	[24]

Endostatin	Endothelial proliferation and migration. Upregulation of endothelial apoptosis. Inhibition of MMPS and bFGF-mediated and VEGF-mediated angiogenesis.	DPSCs, SCAPs, and SHEDs	[16, 30]

IGFBP3	Endothelial migration and tube formation. Inhibition of MMP9 and VEGF production.	DPSCs, SCAPs, and FSCs	[24, 26, 30]

MMPs	Inhibition of FGFR1 and uPAR-mediated signaling. Generation of angiogenic inhibitors by proteolytic cleavage.	DPSCs	[17]

Pentraxin-3 (PTX-3)	Inhibition of bFGF-mediated angiogenesis.	DPSCs, SCAPs, and FSCs	[24, 26]

Pigment epithelium-derived factor (PEGF)	Endothelial proliferation and migration. Upregulation of endothelial apoptosis. Inhibition of MMPs and bFGF-mediated and VEGF-mediated angiogenesis.	DPSCs, SCAPs, and FSCs	[24, 26]

Plasminogen activator inhibitor (PAI-1)	Inhibition of uPA.	DPSCs, SCAPs, and FSCs	[24, 26, 30]

Thrombospondin-1 (THBS1)	Endothelial proliferation, migration, and tube formation. Upregulation of endothelial apoptosis.	DPSCs, SCAPs, and FSCs	[24, 26]

Tissue inhibitor of MMPs-1/4 (TIMP-1/4)	Inhibition of MMPs	DPSCs, SCAPs, and FSCs	[24, 26, 30]

*Neurotrophic factors*			

Basic fibroblast growth factor (bFGF)	Neuronal differentiation. Neurite outgrowth.	DPSCs, SCAPs, SHEDs, and PDLSCs	[16, 26–32]

Brain-derived neurotrophic factor (BDNF)	Survival of neurons. Differentiation of neuroblasts. Formation of synapses and neuritogenesis.	DPSCs, SCAP	[37–41]

Ciliary neurotrophic factor (CNTF)	Neuronal survival.	DPSCs	[40]

Glial-cell derived neurotrophic factor (GDNF)	Survival of neurons. Differentiation of neuroblasts. Neuritogenesis.	DPSCs, SCAPs	[37, 38]

Nerve growth factor (NGF)	Survival, maintenance, and proliferation of neurons. Neurite outgrowth.	DPSCs, SCAPs	[37, 38, 40, 41]

Neurotrophin-3 (NT-3)	Survival of neurons. Differentiation of neuroblasts. Neuritogenesis.	DPSCs	[38, 40]

Neurotrophin-4 (NT-4)	Survival of neurons. Differentiation of neuroblasts. Neuritogenesis.	DPSCs	[40]

PDGF-AA	Neuronal survival. Neuritogenesis.	DPSCs	[40]

VGF (VGF nerve growth factor inducible)	Neuronal survival. Neuritogenesis.	DPSCs	[40]

**Table 2:** Dental stem cells and the effects of preconditioning.

Priming	(Angiogenic) effect	Reference
*Dental pulp stem cells*		
Hypoxia	Increased proliferation rate Increased HIF-1*α* and VEGF expression/secretion Increased migration	[110, 111] [34] [112]
PHD inhibitors	Increased HIF-1*α* and VEGF expression/secretion	[113]
Hinokitiol	Increased HIF-1*α* and VEGF expression/secretion Increased hemoglobin content in mouse matrigel plug assay	[114]
FGF-2	Enhanced EC capillary network formation	[115]
Lipopolysaccharide (LPS)	Increased VEGF expression	[116, 117]

*Stem cells from human exfoliated deciduous teeth*		
Hypoxia	Increased migration	[112]

*Periodontal ligament stem cells*		
Hypoxia	Increased VEGF expression	[118]
PHD inhibitors	Increased HIF-1*α* and VEGF expression/secretion	[119]
IL-1*α*	Increased VEGF expression	[36]
TNF-*α*	Increased VEGF expression	[120]
Adiponectin	Increased proliferation rate Increased wound healing	[121]

*Follicle stem cells*		
Lipopolysaccharide (LPS)	Increased migration	[122]

*Stem cells from the apical papilla*		
Hypoxia	Increased VEGF expression	[26, 31]
PHD inhibitors (CoCl_2_)	Increased HIF-1*α* and VEGF expression/secretion Enhanced EC capillary network formation	[123]

**Table 3:** Clinical application of DSCs.

Condition	Cell type	Status	Location	Principal investigator	Identifier
Dental implants	Allogenic DPSCs	Recruiting	India	Mohammed Sufath UR Rehman Venkat Aditya	NCT02731586
Periodontal disease	Allogenic DPSCs	Recruiting	China	Songlin Wang	NCT02523651
Pulp necrosis	Autologous SHEDs	Recruiting	China	Songtao Shi	NCT01814436
Stroke	Autologous DPSCs	/	Australia	Simon Koblar	TBA

## References

[1] Langer R., Vacanti J. P. (1993). Tissue engineering. *Science*.

[2] Erices A., Conget P., Minguell J. J. (2000). Mesenchymal progenitor cells in human umbilical cord blood. *British Journal of Haematology*.

[3] Friedenstein A. J. (1961). Osteogenetic activity of transplanted transitional epithelium. *Acta Anatomica*.

[4] Gronthos S., Mankani M., Brahim J., Robey P. G., Shi S. (2000). Postnatal human dental pulp stem cells (DPSCs) in vitro and in vivo. *Proceedings of the National Academy of Sciences of the United States of America*.

[5] Mitchell K. E., Weiss M. L., Mitchell B. M. (2003). Matrix cells from Wharton's jelly form neurons and glia. *STEM CELLS*.

[6] Zuk P. A., Zhu M., Mizuno H. (2001). Multilineage cells from human adipose tissue: implications for cell-based therapies. *Tissue Engineering*.

[7] Thesleff I., Nieminen P. (1996). Tooth morphogenesis and cell differentiation. *Current Opinion in Cell Biology*.

[8] Thesleff I., Vaahtokari A., Kettunen P., Aberg T. (1995). Epithelial-mesenchymal signaling during tooth development. *Connective Tissue Research*.

[9] Miura M., Gronthos S., Zhao M. (2003). SHED: stem cells from human exfoliated deciduous teeth. *Proceedings of the National Academy of Sciences of the United States of America*.

[10] Sonoyama W., Liu Y., Yamaza T. (2008). Characterization of the apical papilla and its residing stem cells from human immature permanent teeth: a pilot study. *Journal of Endodontics*.

[11] Morsczeck C., Götz W., Schierholz J. (2005). Isolation of precursor cells (PCs) from human dental follicle of wisdom teeth. *Matrix Biology*.

[12] Seo B.-M., Miura M., Gronthos S. (2004). Investigation of multipotent postnatal stem cells from human periodontal ligament. *The Lancet*.

[13] Dominici M., Le Blanc K., Mueller I. (2006). Minimal criteria for defining multipotent mesenchymal stromal cells. The International Society for Cellular Therapy position statement. *Cytotherapy*.

[14] Gronthos S., Brahim J., Li W. (2002). Stem cell properties of human dental pulp stem cells. *Journal of Dental Research*.

[15] Hilkens P., Gervois P., Fanton Y. (2013). Effect of isolation methodology on stem cell properties and multilineage differentiation potential of human dental pulp stem cells. *Cell and Tissue Research*.

[16] Huang G. T.-J., Gronthos S., Shi S. (2009). Critical reviews in oral biology & medicine: mesenchymal stem cells derived from dental tissues vs. those from other sources: their biology and role in Regenerative Medicine. *Journal of Dental Research*.

[17] Nakashima M., Iohara K., Sugiyama M. (2009). Human dental pulp stem cells with highly angiogenic and neurogenic potential for possible use in pulp regeneration. *Cytokine and Growth Factor Reviews*.

[18] Nuti N., Corallo C., Chan B. M., Ferrari M., Gerami-Naini B. (2016). Multipotent differentiation of human dental pulp stem cells: a literature review. *Stem Cell Reviews and Reports*.

[19] Pierdomenico L., Bonsi L., Calvitti M. (2005). Multipotent mesenchymal stem cells with immunosuppressive activity can be easily isolated from dental pulp. *Transplantation*.

[20] Sharpe P. T. (2016). Dental mesenchymal stem cells. *Development*.

[21] Ding G., Niu J., Wei F. (2015). Current understanding of orofacial tissue derived mesenchymal stem cells: an immunological perspective. *Histology and Histopathology*.

[22] Kaukua N., Shahidi M. K., Konstantinidou C. (2014). Glial origin of mesenchymal stem cells in a tooth model system. *Nature*.

[23] Hilkens P., Meschi N., Lambrechts P., Bronckaers A., Lambrichts I. (2015). Dental stem cells in pulp regeneration: near future or long road ahead?. *Stem Cells and Development*.

[24] Hilkens P., Fanton Y., Martens W. (2014). Pro-angiogenic impact of dental stem cells in vitro and in vivo. *Stem Cell Research*.

[25] Yu S., Zhao Y., Ma Y., Ge L. (2016). Profiling the secretome of human stem cells from dental apical papilla. *Stem Cells and Development*.

[26] Bakopoulou A., Kritis A., Andreadis D. (2015). Angiogenic potential and secretome of human apical papilla mesenchymal stem cells in various stress microenvironments. *Stem Cells and Development*.

[27] Osman A., Gnanasegaran N., Govindasamy V. (2014). Basal expression of growth-factor-associated genes in periodontal ligament stem cells reveals multiple distinctive pathways. *International Endodontic Journal*.

[28] Tran-Hung L., Laurent P., Camps J., About I. (2008). Quantification of angiogenic growth factors released by human dental cells after injury. *Archives of Oral Biology*.

[29] Tran-Hung L., Mathieu S., About I. (2006). Role of human pulp fibroblasts in angiogenesis. *Journal of Dental Research*.

[30] Bronckaers A., Hilkens P., Fanton Y. (2013). Angiogenic properties of human dental pulp stem cells. *PLoS ONE*.

[31] Vanacker J., Viswanath A., De Berdt P. (2014). Hypoxia modulates the differentiation potential of stem cells of the apical papilla. *Journal of Endodontics*.

[32] Yeasmin S., Ceccarelli J., Vigen M. (2014). Stem cells derived from tooth periodontal ligament enhance functional angiogenesis by endothelial cells. *Tissue Engineering—Part A*.

[33] Gorin C., Rochefort G. Y., Bascetin R. (2016). Priming dental pulp stem cells with fibroblast growth factor-2 increases angiogenesis of implanted tissue-engineered constructs through hepatocyte growth factor and vascular endothelial growth factor secretion. *Stem Cells Translational Medicine*.

[34] Aranha A. M. F., Zhang Z., Neiva K. G., Costa C. A. S., Hebling J., Nör J. E. (2010). Hypoxia enhances the angiogenic potential of human dental pulp cells. *Journal of Endodontics*.

[35] Matsushita K., Motani R., Sakuta T. (2000). The role of vascular endothelial growth factor in human dental pulp cells: induction of chemotaxis, proliferation, and differentiation and activation of the AP-1-dependent signaling pathway. *Journal of Dental Research*.

[36] Bando Y., Noguchi K., Kobayashi H., Yoshida N., Ishikawa I., Izumi Y. (2009). Cyclooxygenase-2-derived prostaglandin E2 is involved in vascular endothelial growth factor production in interleukin-1*α*-stimulated human periodontal ligament cells. *Journal of Periodontal Research*.

[37] De Almeida J. F., Chen P., Henry M. A., Diogenes A. (2014). Stem cells of the apical papilla regulate trigeminal neurite outgrowth and targeting through a BDNF-dependent mechanism. *Tissue Engineering—Part A*.

[38] Martens W., Sanen K., Georgiou M. (2014). Human dental pulp stem cells can differentiate into Schwann cells and promote and guide neurite outgrowth in an aligned tissue-engineered collagen construct in vitro. *The FASEB Journal*.

[39] Mead B., Logan A., Berry M., Leadbeater W., Scheven B. A. (2013). Intravitreally transplanted dental pulp stem cells promote neuroprotection and axon regeneration of retinal ganglion cells after optic nerve injury. *Investigative Ophthalmology & Visual Science*.

[40] Mead B., Logan A., Berry M., Leadbeater W., Scheven B. A. (2014). Paracrine-mediated neuroprotection and neuritogenesis of axotomised retinal ganglion cells by human dental pulp stem cells: comparison with human bone marrow and adipose-derived mesenchymal stem cells. *PLoS ONE*.

[41] Nesti C., Pardini C., Barachini S. (2011). Human dental pulp stem cells protect mouse dopaminergic neurons against MPP+ or rotenone. *Brain Research*.

[42] Carmeliet P. (2000). Mechanisms of angiogenesis and arteriogenesis. *Nature Medicine*.

[43] Folkman J. (1971). Tumor angiogenesis: therapeutic implications. *The New England Journal of Medicine*.

[44] Distler J. H. W., Hirth A., Kurowska-Stolarska M., Gay R. E., Gay S., Distler O. (2003). Angiogenic and angiostatic factors in the molecular control of angiogenesis. *Quarterly Journal of Nuclear Medicine*.

[45] Bhadada S. V., Goyal B. R., Patel M. M. (2011). Angiogenic targets for potential disorders. *Fundamental and Clinical Pharmacology*.

[46] Ferrara N., Kerbel R. S. (2005). Angiogenesis as a therapeutic target. *Nature*.

[47] Staudacher D. L., Flugelman M. Y. (2006). Cell and gene therapies in cardiovascular disease with special focus on the no option patient. *Current Gene Therapy*.

[48] Giordano A., Galderisi U., Marino I. R. (2007). From the laboratory bench to the patient's bedside: an update on clinical trials with mesenchymal stem cells. *Journal of Cellular Physiology*.

[49] Psaltis P. J., Zannettino A. C. W., Worthley S. G., Gronthos S. (2008). Concise review: mesenchymal stromal cells: potential for cardiovascular repair. *STEM CELLS*.

[50] Sieveking D. P., Ng M. K. C. (2009). Cell therapies for therapeutic angiogenesis: back to the bench. *Vascular Medicine*.

[51] Guo L., Li J., Qiao X. (2013). Comparison of odontogenic differentiation of human dental follicle cells and human dental papilla cells. *PLoS ONE*.

[52] Liekens S., De Clercq E., Neyts J. (2001). Angiogenesis: regulators and clinical applications. *Biochemical Pharmacology*.

[53] Iohara K., Zheng L., Wake H. (2008). A novel stem cell source for vasculogenesis in ischemia: subfraction of side population cells from dental pulp. *Stem Cells*.

[54] Yuan C., Wang P., Zhu L. (2015). Coculture of stem cells from apical papilla and human umbilical vein endothelial cell under hypoxia increases the formation of three-dimensional vessel-like structures in vitro. *Tissue Engineering Part A*.

[55] Dissanayaka W. L., Zhan X., Zhang C., Hargreaves K. M., Jin L., Tong E. H. Y. (2012). Coculture of dental pulp stem cells with endothelial cells enhances osteo-/odontogenic and angiogenic potential in vitro. *Journal of Endodontics*.

[56] Janebodin K., Zeng Y., Buranaphatthana W., Ieronimakis N., Reyes M. (2013). VEGFR2-dependent angiogenic capacity of pericyte-like dental pulp stem cells. *Journal of Dental Research*.

[57] Iwasaki K., Komaki M., Yokoyama N. (2013). Periodontal ligament stem cells possess the characteristics of pericytes. *Journal of Periodontology*.

[58] Dissanayaka W. L., Hargreaves K. M., Jin L., Samaranayake L. P., Zhang C. (2015). The interplay of dental pulp stem cells and endothelial cells in an injectable peptide hydrogel on angiogenesis and pulp regeneration in vivo. *Tissue Engineering—Part A*.

[59] Gandia C., Armiñan A. N. A., García-Verdugo J. M. (2008). Human dental pulp stem cells improve left ventricular function, induce angiogenesis, and reduce infarct size in rats with acute myocardial infarction. *Stem Cells*.

[60] Ishizaka R., Hayashi Y., Iohara K. (2013). Stimulation of angiogenesis, neurogenesis and regeneration by side population cells from dental pulp. *Biomaterials*.

[61] Sugiyama M., Iohara K., Wakita H. (2011). Dental pulp-derived CD31^−^/CD146^−^ side population stem/progenitor cells enhance recovery of focal cerebral ischemia in rats. *Tissue Engineering Part A*.

[62] d'Aquino R., Graziano A., Sampaolesi M. (2007). Human postnatal dental pulp cells co-differentiate into osteoblasts and endotheliocytes: a pivotal synergy leading to adult bone tissue formation. *Cell Death & Differentiation*.

[63] Marchionni C., Bonsi L., Alviano F. (2009). Angiogenic potential of human dental pulp stromal (stem) cells. *International Journal of Immunopathology and Pharmacology*.

[64] Barachini S., Danti S., Pacini S. (2014). Plasticity of human dental pulp stromal cells with bioengineering platforms: a versatile tool for regenerative medicine. *Micron*.

[65] Karbanová J., Soukup T., Suchánek J., Pytlík R., Corbeil D., Mokrý J. (2011). Characterization of dental pulp stem cells from impacted third molars cultured in low serum-containing medium. *Cells Tissues Organs*.

[66] Cordeiro M. M., Dong Z., Kaneko T. (2008). Dental pulp tissue engineering with stem cells from exfoliated deciduous teeth. *Journal of Endodontics*.

[67] Bento L. W., Zhang Z., Imai A. (2013). Endothelial differentiation of SHED requires MEK1/ERK signaling. *Journal of Dental Research*.

[68] Sakai V. T., Zhang Z., Dong Z. (2010). SHED differentiate into functional odontoblasts and endothelium. *Journal of Dental Research*.

[69] Zhang Z., Nör F., Oh M., Cucco C., Shi S., Nör J. E. (2016). Wnt/*β*-catenin signaling determines the vasculogenic fate of postnatal mesenchymal stem cells. *Stem Cells*.

[70] Amorim B. R., Silvério K. G., Casati M. Z., Sallum E. A., Kantovitz K. R., Nociti F. H. (2016). Neuropilin controls endothelial differentiation by mesenchymal stem cells from the periodontal ligament. *Journal of Periodontology*.

[71] Barros M. A., Martins J. F., Maria D. A. (2015). Immature dental pulp stem cells showed renotropic and pericyte-like properties in acute renal failure in rats. *Cell Medicine*.

[72] Shi S., Gronthos S. (2003). Perivascular niche of postnatal mesenchymal stem cells in human bone marrow and dental pulp. *Journal of Bone and Mineral Research*.

[73] Soria J. M., Sancho-Tello M., Esparza M. A. G. (2011). Biomaterials coated by dental pulp cells as substrate for neural stem cell differentiation. *Journal of Biomedical Materials Research Part A*.

[74] Martens W., Bronckaers A., Politis C., Jacobs R., Lambrichts I. (2013). Dental stem cells and their promising role in neural regeneration: an update. *Clinical Oral Investigations*.

[75] Nosrat I. V., Widenfalk J., Olson L., Nosrat C. A. (2001). Dental pulp cells produce neurotrophic factors, interact with trigeminal neurons in vitro, and rescue motoneurons after spinal cord injury. *Developmental Biology*.

[76] Arthur A., Rychkov G., Shi S., Koblar S. A., Gronthose S. (2008). Adult human dental pulp stem cells differentiate toward functionally active neurons under appropriate environmental cues. *Stem Cells*.

[77] Apel C., Forlenza O. V., De Paula V. J. R. (2009). The neuroprotective effect of dental pulp cells in models of Alzheimer's and Parkinson's disease. *Journal of Neural Transmission*.

[78] Gervois P., Struys T., Hilkens P. (2015). Neurogenic maturation of human dental pulp stem cells following neurosphere generation induces morphological and electrophysiological characteristics of functional neurons. *Stem Cells and Development*.

[79] Nosrat I. V., Smith C. A., Mullally P., Olson L., Nosrat C. A. (2004). Dental pulp cells provide neurotrophic support for dopaminergic neurons and differentiate into neurons in vitro; implications for tissue engineering and repair in the nervous system. *European Journal of Neuroscience*.

[80] Mead B., Logan A., Berry M., Leadbeater W., Scheven B. A. (2014). Dental pulp stem cells, a paracrine-mediated therapy for the retina. *Neural Regeneration Research*.

[81] Arthur A., Shi S., Zannettino A. C. W., Fujii N., Gronthos S., Koblar S. A. (2009). Implanted adult human dental pulp stem cells induce endogenous axon guidance. *STEM CELLS*.

[82] Sugimura-Wakayama Y., Katagiri W., Osugi M. (2015). Peripheral nerve regeneration by secretomes of stem cells from human exfoliated deciduous teeth. *Stem Cells and Development*.

[83] Jarmalavičiute A., Tunaitis V., Pivoraite U., Venalis A., Pivoriunas A. (2015). Exosomes from dental pulp stem cells rescue human dopaminergic neurons from 6-hydroxy-dopamine-induced apoptosis. *Cytotherapy*.

[84] Song M., Jue S.-S., Cho Y.-A., Kim E.-C. (2015). Comparison of the effects of human dental pulp stem cells and human bone marrow-derived mesenchymal stem cells on ischemic human astrocytes in vitro. *Journal of Neuroscience Research*.

[85] Király M., Kádár K., Horváthy D. B. (2011). Integration of neuronally predifferentiated human dental pulp stem cells into rat brain in vivo. *Neurochemistry International*.

[86] Sakai K., Yamamoto A., Matsubara K. (2012). Human dental pulp-derived stem cells promote locomotor recovery after complete transection of the rat spinal cord by multiple neuro-regenerative mechanisms. *The Journal of Clinical Investigation*.

[87] Leong W. K., Henshall T. L., Arthur A. (2012). Human adult dental pulp stem cells enhance poststroke functional recovery through non-neural replacement mechanisms. *Stem Cells Translational Medicine*.

[88] Yang K.-L., Chen M.-F., Liao C.-H., Pang C.-Y., Lin P.-Y. (2009). A simple and efficient method for generating Nurr1-positive neuronal stem cells from human wisdom teeth (tNSC) and the potential of tNSC for stroke therapy. *Cytotherapy*.

[89] Yamagata M., Yamamoto A., Kako E. (2013). Human dental pulp-derived stem cells protect against hypoxic-ischemic brain injury in neonatal mice. *Stroke*.

[90] Tseng L.-S., Chen S.-H., Lin M.-T., Lin Y.-C. (2015). Transplantation of human dental pulp-derived stem cells protects against heatstroke in mice. *Cell Transplantation*.

[91] Nagpal A., Kremer K. L., Hamilton-Bruce M. A. (2016). TOOTH (The Open study Of dental pulp stem cell Therapy in Humans): study protocol for evaluating safety and feasibility of autologous human adult dental pulp stem cell therapy in patients with chronic disability after stroke. *International Journal of Stroke*.

[92] Fujii H., Matsubara K., Sakai K. (2015). Dopaminergic differentiation of stem cells from human deciduous teeth and their therapeutic benefits for Parkinsonian rats. *Brain Research*.

[93] Shimojima C., Takeuchi H., Jin S. (2016). Conditioned medium from the stem cells of human exfoliated deciduous teeth ameliorates experimental autoimmune encephalomyelitis. *The Journal of Immunology*.

[94] De Berdt P., Vanacker J., Ucakar B. (2015). Dental apical papilla as therapy for spinal cord injury. *Journal of Dental Research*.

[95] Li X., Yang C., Li L. (2015). A therapeutic strategy for spinal cord defect: human dental follicle cells combined with aligned PCL/PLGA electrospun material. *BioMed Research International*.

[96] Heng B. C., Lim L. W., Wu W., Zhang C. (2016). An overview of protocols for the neural induction of dental and oral stem cells in vitro. *Tissue Engineering Part B: Reviews*.

[97] Askari N., Yaghoobi M. M., Shamsara M., Esmaeili-Mahani S. (2014). Human dental pulp stem cells differentiate into oligodendrocyte progenitors using the expression of Olig2 transcription factor. *Cells Tissues Organs*.

[98] Deumens R., Bozkurt A., Meek M. F. (2010). Repairing injured peripheral nerves: bridging the gap. *Progress in Neurobiology*.

[99] Gaudet A. D., Popovich P. G., Ramer M. S. (2011). Wallerian degeneration: gaining perspective on inflammatory events after peripheral nerve injury. *Journal of Neuroinflammation*.

[100] Hall S. M. (1986). The effect of inhibiting Schwann cell mitosis on the re-innervation of acellular autografts in the peripheral nervous system of the mouse. *Neuropathology and Applied Neurobiology*.

[101] Sasaki R., Aoki S., Yamato M. (2011). PLGA artificial nerve conduits with dental pulp cells promote facial nerve regeneration. *Journal of Tissue Engineering and Regenerative Medicine*.

[102] Sasaki R., Aoki S., Yamato M. (2008). Tubulation with dental pulp cells promotes facial nerve regeneration in rats. *Tissue Engineering Part A*.

[103] Sasaki R., Matsumine H., Watanabe Y. (2014). Electrophysiologic and functional evaluations of regenerated facial nerve defects with a tube containing dental pulp cells in rats. *Plastic and Reconstructive Surgery*.

[104] Li B. H., Jung H.-J., Kim S.-M., Kim M.-J., Jahng J. W., Lee J.-H. (2013). Human periodontal ligament stem cells repair mental nerve injury. *Neural Regeneration Research*.

[105] Muscari C., Giordano E., Bonafè F., Govoni M., Pasini A., Guarnieri C. (2013). Priming adult stem cells by hypoxic pretreatments for applications in regenerative medicine. *Journal of Biomedical Science*.

[106] Li W., Ma N., Ong L.-L. (2007). Bcl-2 engineered MSCs inhibited apoptosis and improved heart function. *Stem Cells*.

[107] Noiseux N., Gnecchi M., Lopez-Ilasaca M. (2006). Mesenchymal stem cells overexpressing Akt dramatically repair infarcted myocardium and improve cardiac function despite infrequent cellular fusion or differentiation. *Molecular Therapy*.

[108] Gnecchi M., He H., Noiseux N. (2006). Evidence supporting paracrine hypothesis for Akt-modified mesenchymal stem cell-mediated cardiac protection and functional improvement. *The FASEB Journal*.

[109] Phillips M. I., Tang Y. L. (2008). Genetic modification of stem cells for transplantation. *Advanced Drug Delivery Reviews*.

[110] Iida K., Takeda-Kawaguchi T., Tezuka Y., Kunisada T., Shibata T., Tezuka K.-I. (2010). Hypoxia enhances colony formation and proliferation but inhibits differentiation of human dental pulp cells. *Archives of Oral Biology*.

[111] Sakdee J. B., White R. R., Pagonis T. C., Hauschka P. V. (2009). Hypoxia-amplified proliferation of human dental pulp cells. *Journal of Endodontics*.

[112] Kanafi M. M., Ramesh A., Gupta P. K., Bhonde R. R. (2013). Influence of hypoxia, high glucose, and low serum on the growth kinetics of mesenchymal stem cells from deciduous and permanent teeth. *Cells Tissues Organs*.

[113] Müller H.-D., Cvikl B., Gruber R., Watzek G., Agis H. (2012). Prolyl hydroxylase inhibitors increase the production of vascular endothelial growth factor in dental pulp-derived cells. *Journal of Endodontics*.

[114] Kim M.-K., Park H.-J., Kim Y.-D. (2014). Hinokitiol increases the angiogenic potential of dental pulp cells through ERK and p38MAPK activation and hypoxia-inducible factor-1*α* (HIF-1*α*) upregulation. *Archives of Oral Biology*.

[115] Kim Y.-S., Min K.-S., Jeong D.-H., Jang J.-H., Kim H.-W., Kim E.-C. (2010). Effects of fibroblast growth factor-2 on the expression and regulation of chemokines in human dental pulp cells. *Journal of Endodontics*.

[116] Matsushita K., Motani R., Sakuta T. (1999). Lipopolysaccharide enhances the production of vascular endothelial growth factor by human pulp cells in culture. *Infection and Immunity*.

[117] Botero T. M., Mantellini M. G., Song W., Hanks C. T., Nör J. E. (2003). Effect of lipopolysaccharides on vascular endothelial growth factor expression in mouse pulp cells and macrophages. *European Journal of Oral Sciences*.

[118] Motohira H., Hayashi J., Tatsumi J., Tajima M., Sakagami H., Shin K. (2007). Hypoxia and reoxygenation augment bone-resorbing factor production from human periodontal ligament cells. *Journal of Periodontology*.

[119] Agis H., Watzek G., Gruber R. (2012). Prolyl hydroxylase inhibitors increase the production of vascular endothelial growth factor by periodontal fibroblasts. *Journal of Periodontal Research*.

[120] Oyama T., Sakuta T., Matsushita K., Maruyama I., Nagaoka S., Torii M. (2000). Effects of roxithromycin on tumor necrosis factor-alpha-induced vascular endothelial growth factor expression in human periodontal ligament cells in culture. *Journal of Periodontology*.

[121] Nokhbehsaim M., Keser S., Nogueira A. V. B. (2014). Beneficial effects of adiponectin on periodontal ligament cells under normal and regenerative conditions. *Journal of Diabetes Research*.

[122] Chatzivasileiou K., Lux C. A., Steinhoff G., Lang H. (2013). Dental follicle progenitor cells responses to Porphyromonas gingivalis LPS. *Journal of Cellular and Molecular Medicine*.

[123] Yuan C., Wang P., Zhu L. (2015). Coculture of stem cells from apical papilla and human umbilical vein endothelial cell under hypoxia increases the formation of three-dimensional vessel-like structures in vitro. *Tissue Engineering—Part A*.

[124] Baraniak P. R., McDevitt T. C. (2010). Stem cell paracrine actions and tissue regeneration. *Regenerative Medicine*.

[125] Rosová I., Dao M., Capoccia B., Link D., Nolta J. A. (2008). Hypoxic preconditioning results in increased motility and improved therapeutic potential of human mesenchymal stem cells. *Stem Cells*.

[126] Kubo M., Li T.-S., Suzuki R. (2008). Hypoxic preconditioning increases survival and angiogenic potency of peripheral blood mononuclear cells via oxidative stress resistance. *American Journal of Physiology—Heart and Circulatory Physiology*.

[127] Potier E., Ferreira E., Andriamanalijaona R. (2007). Hypoxia affects mesenchymal stromal cell osteogenic differentiation and angiogenic factor expression. *Bone*.

[128] Huang G. T., Al-Habib M., Gauthier P. (2013). Challenges of stem cell-based pulp and dentin regeneration: a clinical perspective. *Endodontic Topics*.

[129] Lee M. J., Kim J. W., Yang E. G. (2010). Hinokitiol activates the hypoxia-inducible factor (HIF) pathway through inhibition of HIF hydroxylases. *Biochemical and Biophysical Research Communications*.

[130] Trimmel K., Cvikl B., Müller H.-D. (2015). L-mimosine increases the production of vascular endothelial growth factor in human tooth slice organ culture model. *International Endodontic Journal*.

[131] Wei L., Fraser J. L., Lu Z.-Y., Hu X., Yu S. P. (2012). Transplantation of hypoxia preconditioned bone marrow mesenchymal stem cells enhances angiogenesis and neurogenesis after cerebral ischemia in rats. *Neurobiology of Disease*.

[132] Wei F., Song T., Ding G. (2013). Functional tooth restoration by allogeneic mesenchymal stem cell-based bio-root regeneration in swine. *Stem Cells and Development*.

[133] Kim Y. S., Noh M. Y., Cho K. A. (2015). Hypoxia/reoxygenation-preconditioned human bone marrow-derived mesenchymal stromal cells rescue ischemic rat cortical neurons by enhancing trophic factor release. *Molecular Neurobiology*.

[134] Theus M. H., Wei L., Cui L. (2008). In vitro hypoxic preconditioning of embryonic stem cells as a strategy of promoting cell survival and functional benefits after transplantation into the ischemic rat brain. *Experimental Neurology*.

[135] Hargreaves K. M., Diogenes A., Teixeira F. B. (2013). Treatment options: biological basis of regenerative endodontic procedures. *Journal of Endodontics*.

[136] Huang G. T.-J. (2008). A paradigm shift in endodontic management of immature teeth: conservation of stem cells for regeneration. *Journal of Dentistry*.

[137] Schmalz G., Smith A. J. (2014). Pulp development, repair, and regeneration: challenges of the transition from traditional dentistry to biologically based therapies. *Journal of Endodontics*.

[138] Mooney D. J., Powell C., Piana J., Rutherford B. (1996). Engineering dental pulp-like tissue in vitro. *Biotechnology Progress*.

[139] Buurma B., Gu K., Rutherford R. B. (1999). Transplantation of human pulpal and gingival fibroblasts attached to synthetic scaffolds. *European Journal of Oral Sciences*.

[140] Batouli S., Miura M., Brahim J. (2003). Comparison of stem-cell-mediated osteogenesis and dentinogenesis. *Journal of Dental Research*.

[141] Sakai V. T., Cordeiro M. M., Dong Z., Zhang Z., Zeitlin B. D., Nör J. E. (2011). Tooth slice/scaffold model of dental pulp tissue engineering. *Advances in Dental Research*.

[142] Prescott R. S., Alsanea R., Fayad M. I. (2008). In vivo generation of dental pulp-like tissue by using dental pulp stem cells, a collagen scaffold, and dentin matrix protein 1 after subcutaneous transplantation in mice. *Journal of Endodontics*.

[143] Huang G. T.-J., Yamaza T., Shea L. D. (2010). Stem/progenitor cell-mediated de novo regeneration of dental pulp with newly deposited continuous layer of dentin in an *in vivo* model. *Tissue Engineering Part A*.

[144] Rosa V., Zhang Z., Grande R. H. M., Nör J. E. (2013). Dental pulp tissue engineering in full-length human root canals. *Journal of Dental Research*.

[145] Murakami M., Horibe H., Iohara K. (2013). The use of granulocyte-colony stimulating factor induced mobilization for isolation of dental pulp stem cells with high regenerative potential. *Biomaterials*.

[146] Kuang R., Zhang Z., Jin X. (2016). Nanofibrous spongy microspheres for the delivery of hypoxia-primed human dental pulp stem cells to regenerate vascularized dental pulp. *Acta Biomaterialia*.

[147] Iohara K., Nakashima M., Ito M., Ishikawa M., Nakasima A., Akamine A. (2004). Dentin regeneration by dental pulp stem cell therapy with recombinant human bone morphogenetic protein 2. *Journal of Dental Research*.

[148] Iohara K., Zheng L., Ito M., Tomokiyo A., Matsushita K., Nakashima M. (2006). Side population cells isolated from porcine dental pulp tissue with self-renewal and multipotency for dentinogenesis, chondrogenesis, adipogenesis, and neurogenesis. *Stem Cells*.

[149] Iohara K., Fujita M., Ariji Y. (2016). Assessment of pulp regeneration induced by stem cell therapy by magnetic resonance imaging. *Journal of Endodontics*.

[150] Iohara K., Imabayashi K., Ishizaka R. (2011). Complete pulp regeneration after pulpectomy by transplantation of CD105^+^ stem cells with stromal cell-derived factor-1. *Tissue Engineering Part A*.

[151] Iohara K., Murakami M., Nakata K., Nakashima M. (2014). Age-dependent decline in dental pulp regeneration after pulpectomy in dogs. *Experimental Gerontology*.

[152] Iohara K., Murakami M., Takeuchi N. (2013). A novel combinatorial therapy with pulp stem cells and granulocyte colony-stimulating factor for total pulp regeneration. *Stem Cells Translational Medicine*.

[153] Iohara K., Zheng L., Ito M. (2009). Regeneration of dental pulp after pulpotomy by transplantation of CD31^−^/CD146^−^ side population cells from a canine tooth. *Regenerative Medicine*.

[154] Wang Y., Zhao Y., Jia W., Yang J., Ge L. (2013). Preliminary study on dental pulp stem cell-mediated pulp regeneration in canine immature permanent teeth. *Journal of Endodontics*.

[155] Yang J.-W., Zhang Y.-F., Wan C.-Y. (2015). Autophagy in SDF-1*α*-mediated DPSC migration and pulp regeneration. *Biomaterials*.

[156] Ishizaka R., Iohara K., Murakami M., Fukuta O., Nakashima M. (2012). Regeneration of dental pulp following pulpectomy by fractionated stem/progenitor cells from bone marrow and adipose tissue. *Biomaterials*.

[157] Nakashima M., Iohara K. (2014). Mobilized dental pulp stem cells for pulp regeneration: initiation of clinical trial. *Journal of Endodontics*.

[158] Sonoyama W., Liu Y., Fang D. (2006). Mesenchymal stem cell-mediated functional tooth regeneration in Swine. *PLoS ONE*.

[159] Na S., Zhang H., Huang F. (2016). Regeneration of dental pulp/dentine complex with a three-dimensional and scaffold-free stem-cell sheet-derived pellet. *Journal of Tissue Engineering and Regenerative Medicine*.

[160] Bai Y., Bai Y., Matsuzaka K. (2011). Cementum- and periodontal ligament-like tissue formation by dental follicle cell sheets co-cultured with Hertwig's epithelial root sheath cells. *Bone*.

[161] Guo S., Guo W., Yi D. (2013). Comparative study of human dental follicle cell sheets and periodontal ligament cell sheets for periodontal tissue regeneration. *Cell Transplantation*.

[162] Guo W., Chen L., Gong K., Ding B., Duan Y., Jin Y. (2012). Heterogeneous dental follicle cells and the regeneration of complex periodontal tissues. *Tissue Engineering Part A*.

[163] Handa K., Saito M., Tsunoda A. (2002). Progenitor cells from dental follicle are able to form cementum matrix in vivo. *Connective Tissue Research*.

[164] Jung H.-S., Lee D.-S., Lee J.-H. (2011). Directing the differentiation of human dental follicle cells into cementoblasts and/or osteoblasts by a combination of HERS and pulp cells. *Journal of Molecular Histology*.

[165] Oshima M., Inoue K., Nakajima K. (2014). Functional tooth restoration by next-generation bio-hybrid implant as a bio-hybrid artificial organ replacement therapy. *Scientific Reports*.

[166] Sowmya S., Chennazhi K. P., Arzate H., Jayachandran P., Nair S. V., Jayakumar R. (2015). Periodontal specific differentiation of dental follicle stem cells into osteoblast, fibroblast, and cementoblast. *Tissue Engineering Part C: Methods*.

[167] Yokoi T., Saito M., Kiyono T. (2007). Establishment of immortalized dental follicle cells for generating periodontal ligament in vivo. *Cell and Tissue Research*.

[168] Guo W., He Y., Zhang X. (2009). The use of dentin matrix scaffold and dental follicle cells for dentin regeneration. *Biomaterials*.

[169] Guo W., Gong K., Shi H. (2012). Dental follicle cells and treated dentin matrix scaffold for tissue engineering the tooth root. *Biomaterials*.

[170] Yang B., Chen G., Li J. (2012). Tooth root regeneration using dental follicle cell sheets in combination with a dentin matrix—based scaffold. *Biomaterials*.

[171] Jiao L., Xie L., Yang B. (2014). Cryopreserved dentin matrix as a scaffold material for dentin-pulp tissue regeneration. *Biomaterials*.

[172] Ducret M., Fabre H., Degoul O. (2015). Manufacturing of dental pulp cell-based products from human third molars: current strategies and future investigations. *Frontiers in Physiology*.

[173] Perry B. C., Zhou D., Wu X. (2008). Collection, cryopreservation, and characterization of human dental pulp-derived mesenchymal stem cells for banking and clinical use. *Tissue Engineering—Part C: Methods*.

[174] Takeda T., Tezuka Y., Horiuchi M. (2008). Characterization of dental pulp stem cells of human tooth germs. *Journal of Dental Research*.

[175] Eaker S., Armant M., Brandwein H. (2013). Concise review: guidance in developing commercializable autologous/patient-specific cell therapy manufacturing. *Stem Cells Translational Medicine*.

[176] Gottipamula S., Muttigi M. S., Kolkundkar U., Seetharam R. N. (2013). Serum-free media for the production of human mesenchymal stromal cells: a review. *Cell Proliferation*.

[177] Halme D. G., Kessler D. A. (2006). FDA regulation of stem-cell-based therapies. *The New England Journal of Medicine*.

[178] Bieback K. (2013). Platelet lysate as replacement for fetal bovine serum in mesenchymal stromal cell cultures. *Transfusion Medicine and Hemotherapy*.

[179] Burnouf T., Strunk D., Koh M. B., Schallmoser K. (2016). Human platelet lysate: replacing fetal bovine serum as a gold standard for human cell propagation?. *Biomaterials*.

[180] Hemeda H., Giebel B., Wagner W. (2014). Evaluation of human platelet lysate versus fetal bovine serum for culture of mesenchymal stromal cells. *Cytotherapy*.

[181] Ding G., Niu J., Liu Y. (2015). Dental pulp stem cells suppress the proliferation of lymphocytes via transforming growth factor-*β*1. *Human Cell*.

[182] Ding G., Wang W., Liu Y. (2010). Effect of cryopreservation on biological and immunological properties of stem cells from apical papilla. *Journal of Cellular Physiology*.

[183] Ding G., Liu Y., An Y. (2010). Suppression of T cell proliferation by root apical papilla stem cells in vitro. *Cells Tissues Organs*.

[184] Ding G., Liu Y., Wang W. (2010). Allogeneic periodontal ligament stem cell therapy for periodontitis in swine. *Stem Cells*.

[185] Wada N., Menicanin D., Shi S., Bartold P. M., Gronthos S. (2009). Immunomodulatory properties of human periodontal ligament stem cells. *Journal of Cellular Physiology*.

[186] Kim H.-S., Kim K.-H., Kim S.-H. (2010). Immunomodulatory effect of canine periodontal ligament stem cells on allogenic and xenogenic peripheral blood mononuclear cells. *Journal of Periodontal and Implant Science*.

[187] Liu D., Xu J., Liu O. (2012). Mesenchymal stem cells derived from inflamed periodontal ligaments exhibit impaired immunomodulation. *Journal of Clinical Periodontology*.

[188] Li Z., Jiang C.-M., An S. (2014). Immunomodulatory properties of dental tissue-derived mesenchymal stem cells. *Oral Diseases*.

[189] Yildirim S., Zibandeh N., Genc D., Ozcan E. M., Goker K., Akkoc T. (2016). The comparison of the immunologic properties of stem cells isolated from human exfoliated deciduous teeth, dental pulp, and dental follicles. *Stem Cells International*.

[190] Tomic S., Djokic J., Vasilijic S. (2011). Immunomodulatory properties of mesenchymal stem cells derived from dental pulp and dental follicle are susceptible to activation by toll-like receptor agonists. *Stem Cells and Development*.

[191] Zhao Y., Wang L., Jin Y., Shi S. (2012). Fas ligand regulates the immunomodulatory properties of dental pulp stem cells. *Journal of Dental Research*.

[192] Kerkis I., Ambrosio C. E., Kerkis A. (2008). Early transplantation of human immature dental pulp stem cells from baby teeth to golden retriever muscular dystrophy (GRMD) dogs: local or systemic?. *Journal of Translational Medicine*.

[193] Mita T., Furukawa-Hibi Y., Takeuchi H. (2015). Conditioned medium from the stem cells of human dental pulp improves cognitive function in a mouse model of Alzheimer's disease. *Behavioural Brain Research*.

[194] Choi B.-H. (2000). Periodontal ligament formation around titanium implants using cultured periodontal ligament cells: a pilot study. *International Journal of Oral and Maxillofacial Implants*.

[195] Feng F., Akiyama K., Liu Y. (2010). Utility of PDL progenitors for in vivo tissue regeneration: a report of 3 cases. *Oral Diseases*.

[196] Gault P., Black A., Romette J.-L. (2010). Tissue-engineered ligament: implant constructs for tooth replacement. *Journal of Clinical Periodontology*.

[197] Lin Y., Gallucci G. O., Buser D., Bosshardt D., Belser U. C., Yelick P. C. (2011). Bioengineered periodontal tissue formed on titanium dental implants. *Journal of Dental Research*.

[198] Fleming P. S., Koletsi D., O’Brien K., Tsichlaki A., Pandis N. (2016). Are dental researchers asking patient-important questions? a scoping review. *Journal of Dentistry*.

